# A perturbing case of a recurrent dermatofibrosarcoma protuberans and giant cell fibroblastoma hybrid

**DOI:** 10.1016/j.jdcr.2025.04.005

**Published:** 2025-04-21

**Authors:** Chase Andrew Pitchford, Hailey Swan, Joshua Brady, Aaminah Azhar, Sepideh Asadbeigi, Jeffrey McBride, Lindsey Collins

**Affiliations:** Department of Dermatology, OUHSC, Oklahoma City, Oklahoma

**Keywords:** dermatofibrosarcoma protuberans, fibrohistiocytic tumor, giant cell fibroblastoma, hybrid tumor, soft tissue tumor

## Introduction

Dermatofibrosarcoma protuberans (DFSP) is a rare, slow-growing soft tissue sarcoma with a propensity for local aggressiveness and high recurrence rates following surgical excision, despite its low metastatic potential. It primarily originates in the dermis or subcutis and is histologically characterized by storiform spindle cell proliferation, with fibrosarcomatous transformation in some cases contributing to aggressive behavior.^1^ Giant cell fibroblastoma (GCF), another rare tumor, generally behaves in a more indolent manner compared to DFSP and is characterized by a distinct histopathological appearance featuring multinucleated giant cells.^1^^,^^2^ It predominantly affects pediatric patients, with a median age of presentation of 6 years, thus its occurrence in adults is quite rare.^2^

The hybrid form of DFSP and GCF has been documented in a limited number of cases.^3^, ^4^, ^5^ These tumors combine features of DFSP and GCF, posing unique diagnostic challenges due to overlapping histopathological and immunohistochemical profiles and clinical challenges due to risk of recurrence.^6^, ^7^, ^8^ Treatment generally involves surgical excision, with Mohs micrographic surgery (MMS) being favored for its ability to achieve clear margins while preserving tissue. Recurrence rates for DFSP following WLE are reported at 3.7%, as compared to 1.7% for MMS, highlighting its superior margin control and underscoring the importance of meticulous surgical management and long-term surveillance.^4^^,^^9^

We present a case of a hybrid DFSP/GCF lesion in an adult patient, arising on the inguinal skin. This case is further distinguished by its exophytic and pedunculated morphology, a recurrence following complete vulvectomy, and the necessity for multiple stages of MMS to achieve clearance. Additionally, the emergence of a new, distinct lesion in the groin raises concerns about multiple DFSP lesions, contributing to the complexity of this clinical scenario. In presenting this case, we aim to demonstrate a rare complex case of a hybrid DFSP/GCF, discuss the clinical course and treatment challenges, and add to the knowledge of managing such tumors, particularly in adult patients and in atypical anatomical locations.

## Case description

A 51-year-old female with a history of recurrent DFSP presented to the dermatology clinic for evaluation. She initially underwent WLE of a DFSP in 2017, followed by a radical vulvectomy due to recurrence in 2019. Postvulvectomy, a computed tomography scan revealed no evidence of metastatic disease. At the time of presentation, the patient denied weight loss, fever, or chills but reported tenderness in the right inguinal region.

**Fig 1:**
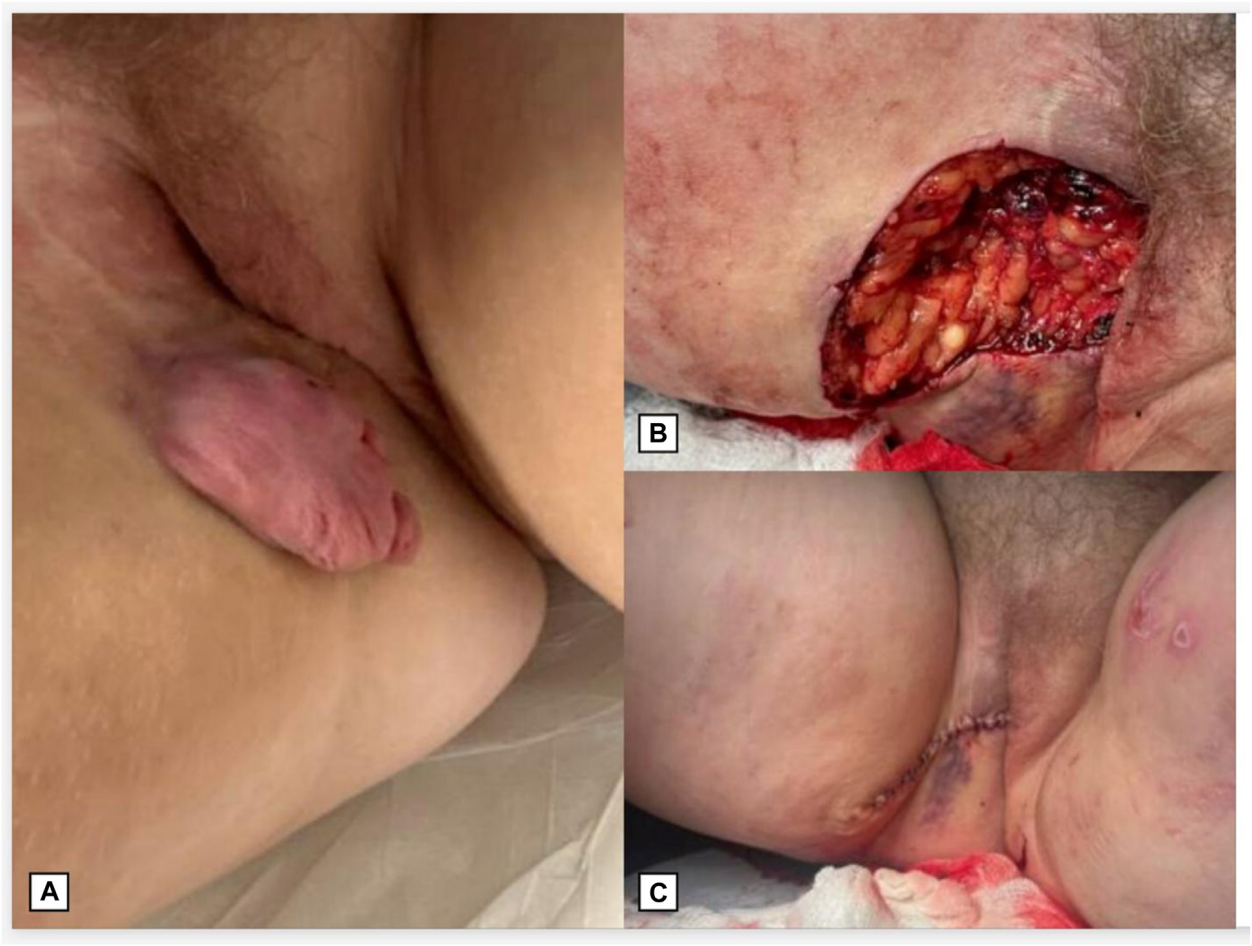
**A,** Clinical presentation of a fleshy, pedunculated, soft plaque measuring 6 cm × 3.5 cm in the right inner thigh within the surgical field of previously treated dermatofibrosarcoma protuberans (DFSP) years prior. **B,** Final surgical defect after 2 stages of slow Mohs surgery, with histologically clear surgical margins. **C,** Intermediate linear closure of final Mohs defect.

On physical examination, a fleshy, pedunculated, soft plaque measuring 6 × 3.5 cm was noted on the right inner thigh within the previous surgical scar (Fig 1, *A*). A biopsy of the lesion was performed, and pathology revealed an atypical epithelioid and hypercellular storiform spindled cell proliferation with scattered giant cells and CD34-positive tumor cells. These features are not the typical features of a DFSP which typically shows solely a storiform pattern of spindled proliferation. Following histopathologic consensus, features were interpreted as recurrent DFSP with hybrid GCF.

**Fig 2:**
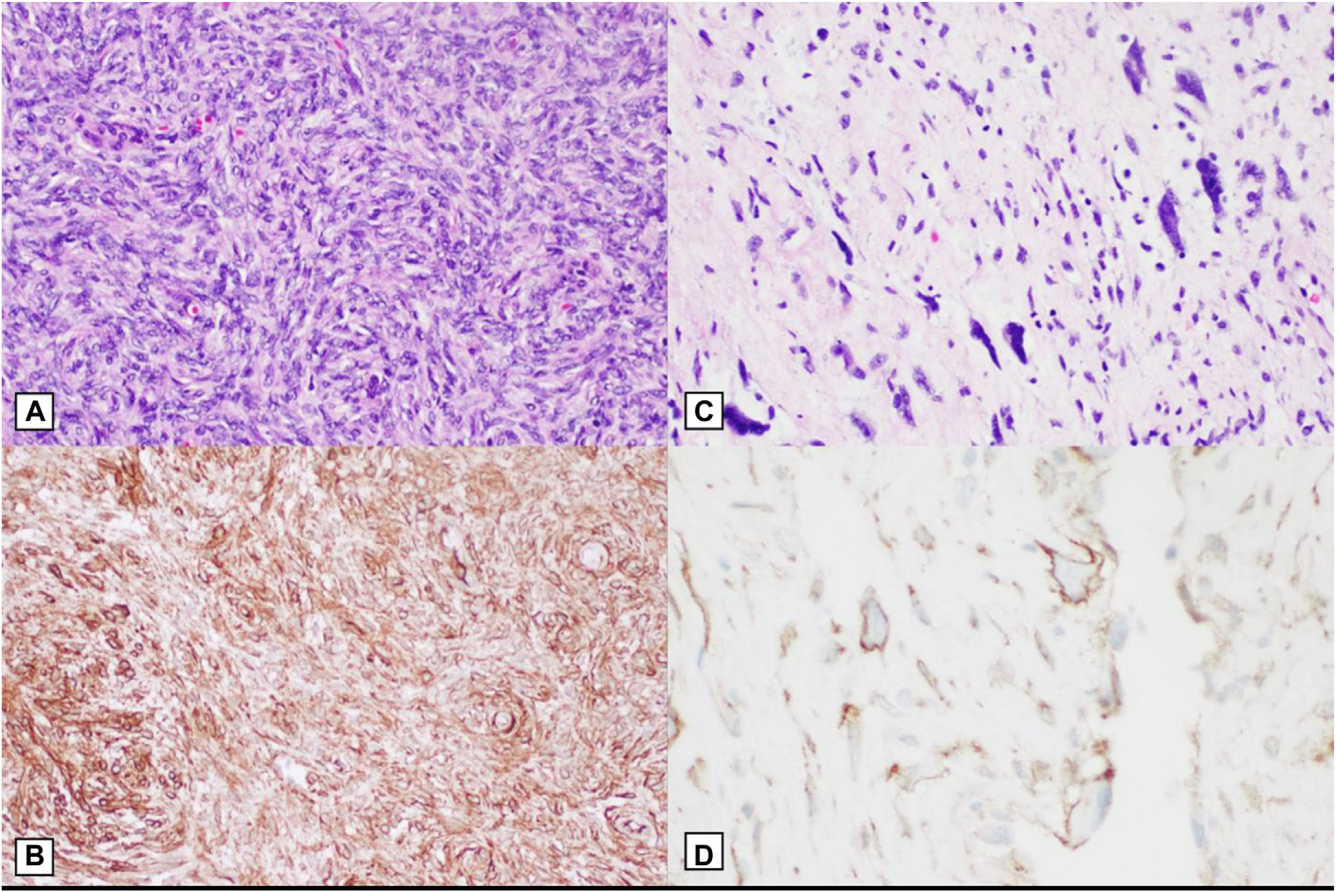
**A,** Hematoxylin and eosin (H&E) staining of a dense, hypercellular storiform spindle cell proliferation in the dermis at 20× magnification. **B,** Immunohistochemistry of CD34 positive spindle cells at 20× magnification. **C,** H&E staining of a dermal population of multinucleated giant cells surrounding pseudovascular spaces in the dermis at 20× magnification. **D,** Immunohistochemistry of CD34 positive giant cells at 40× magnification.

Given the patient’s history of recurrence and the need for high cure rates with optimal functional outcomes, the patient was referred for MMS. She underwent a slow Mohs procedure, which is a variant of MMS performed over several days. The lesion borders were marked, and a 1 cm margin was established. The debulking specimen was excised, extending to the superficial subcutaneous fat. The first Mohs layer was processed for horizontal frozen sections, and both the debulking specimen and peripheral margin were sent for pathology. Pathology from the first Mohs stage confirmed hybrid DFSP and GCF, with CD34 positivity highlighting the tumor cells on all blocks (Fig 2). Due to positive deep margins, the patient returned for a second stage of slow Mohs surgery. The second excision specimen demonstrated clear margins with no residual DFSP, and an intermediate linear closure was performed (Fig 1, *B* and *C*).

**Fig 3:**
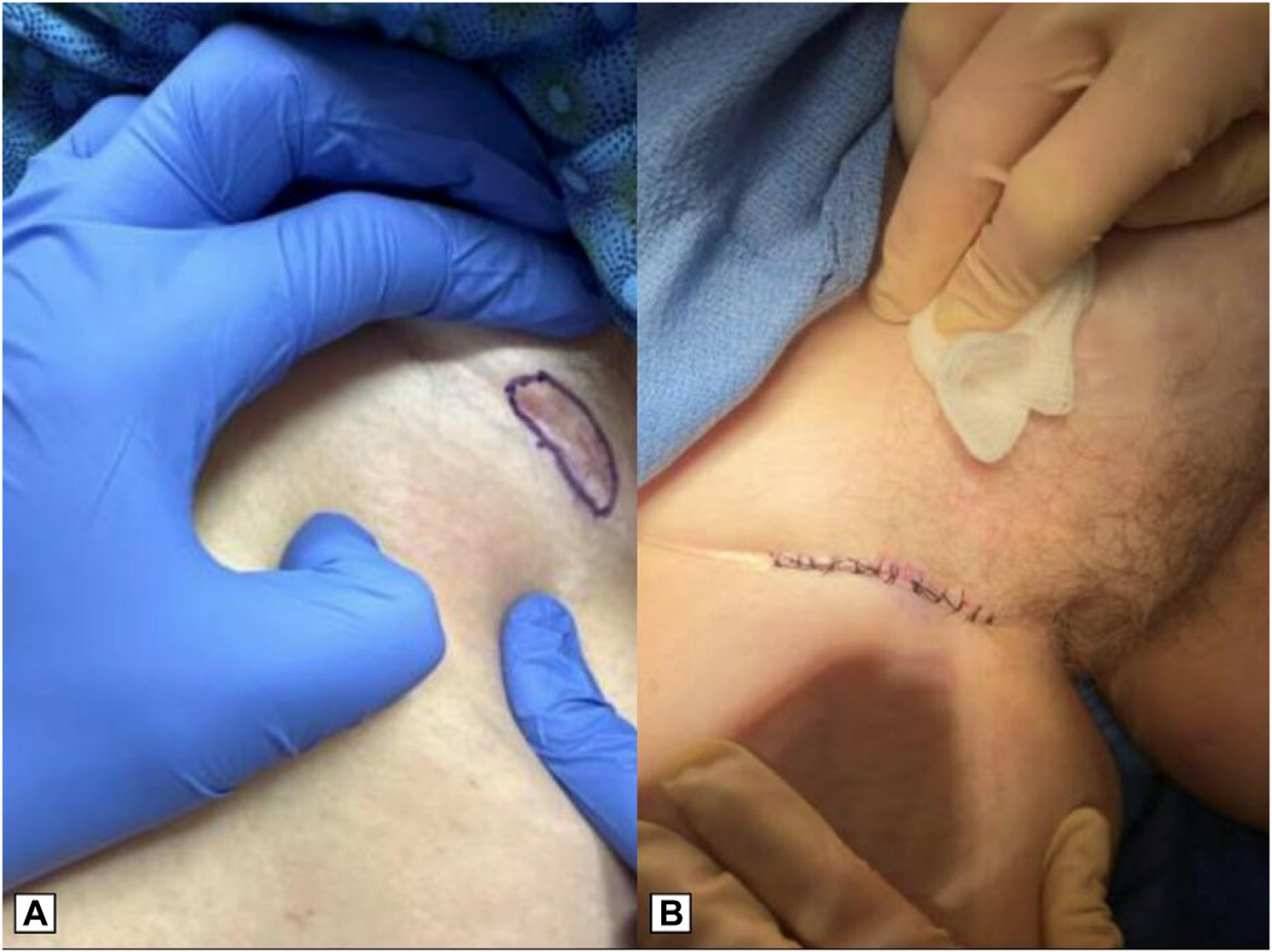
**A,** New, tender plaque in the right inguinal fold, superior to her prior dermatofibrosarcoma protuberans (DFSP) site. **B,** Intermediate linear closure after achievement of clear margins with slow Mohs micrographic surgery.

During her follow-up visit for suture removal, the patient noted the appearance of a new, tender lesion in her right inguinal fold, superior to her prior DFSP surgical site. Examination revealed a 3.0 × 1.0 cm erythematous ill-defined patch with a deeper nodule in the right inguinal fold (Fig 3, *A*). A punch biopsy was performed, and pathology confirmed a CD34-positive spindle cell proliferation, consistent with DFSP. Ki-67 highlighted areas of proliferative index greater than 5% in some foci. Staining with smooth muscle actin was performed, to rule out the possibility of a myofibroblastic component, which was negative. The patient was again referred for slow Mohs surgery for the new lesion, with resultant clear margins after 1 stage and intermediate linear closure of the defect (Fig 3, *B*).

## Discussion/Conclusion

This case highlights several unique and clinically significant aspects of hybrid DFSP/GCF lesions, expanding the limited literature on this rare pathology. These tumors are predominantly reported in pediatric populations, with very few documented occurrences in adults.^4^ Our case is noteworthy for its presentation in an adult patient and its unusual anatomical location in the inguinal skin — an exceedingly rare site for DFSP.^3^^,^^4^^,^^10^

The lesion's recurrence after a complete vulvectomy and prior definitive surgical intervention illustrates the particularly aggressive and persistent nature of this hybrid pathology. The necessity for multiple stages of Mohs surgery to achieve tumor clearance underscores the challenges of managing such lesions in anatomically sensitive areas. This case further supports the utility of Mohs surgery in achieving superior margin control compared to traditional wide local excision, particularly in regions with limited tissue for resection.

The emergence of a new lesion in a separate area of the groin raises the possibility of a second DFSP lesion, a phenomenon that is exceedingly rare and not well-documented in the literature. This finding highlights the importance of rigorous and ongoing surveillance in patients with a history of DFSP or hybrid tumors. By documenting this case, we contribute to the understanding of hybrid DFSP/GCF lesions and provide insights into the clinical behavior, challenges in management, and potential for recurrence. Future research should focus on the underlying biological mechanisms driving the development of these hybrid tumors, refining surveillance strategies, and optimizing treatment protocols to minimize recurrence risks.

## Conflicts of interest

None disclosed.

## References

[1] Sandberg A.A., Bridge J.A. (2003). Updates on the cytogenetics and molecular genetics of bone and soft tissue tumors. Dermatofibrosarcoma protuberans and giant cell fibroblastoma. Cancer Genet Cytogenet.

[2] Jha P., Moosavi C., Fanburg-Smith J.C. (2007). Giant cell fibroblastoma: an update and addition of 86 new cases from the Armed Forces Institute of Pathology, in honor of Dr. Franz M. Enzinger. Ann Diagn Pathol.

[3] Braswell D.S., Ayoubi N., Motaparthi K., Walker A. (2020). Dermatofibrosarcoma protuberans with features of giant cell fibroblastoma in an adult. J Cutan Pathol.

[4] Warbrick-Smith J., Hollowood K., Birch J. (2010). Dermatofibrosarcoma protuberans recurring as a hybrid dermatofibrosarcoma/giant cell fibroblastoma in an adult: a case report. J Plast Reconstr Aesthet Surg.

[5] Michal M., Zamecnik M. (1992). Giant cell fibroblastoma with a dermatofibrosarcoma protuberans component. Am J Dermatopathol.

[6] Harvell J.D., Kilpatrick S.E., White W.L. (1998). Histogenetic relations between giant cell fibroblastoma and dermatofibrosarcoma protuberans. CD34 staining showing the spectrum and a simulator. Am J Dermatopathol.

[7] Beham A., Fletcher C.D. (1990). Dermatofibrosarcoma protuberans with areas resembling giant cell fibroblastoma: report of two cases. Histopathology.

[8] Eminger L.A., Shinohara M.M., Elenitsas R., Halpern A.V., Heymann W.R. (2012). Giant cell fibroblastoma mimicking a soft fibroma arising within a dermatofibrosarcoma protuberans. J Am Acad Dermatol.

[9] Martin E.C.S., Vyas K.S., Batbold S., Erwin P.J., Brewer J.D. (2022). Dermatofibrosarcoma protuberans recurrence after wide local excision versus Mohs micrographic surgery: a systematic review and meta-analysis. Dermatol Surg.

[10] Pascual A., Sánchez-Martínez C., Moreno C., Burdaspal-Moratilla A., López-Rodriguez M.J., Rios L. (2010). Dermatofibrosarcoma protuberans with areas of giant cell fibroblastoma in the vulva: a case report. Eur J Gynaecol Oncol.

